# Density-Dependent Migration Characteristics of Cancer Cells Driven by Pseudopod Interaction

**DOI:** 10.3389/fcell.2022.854721

**Published:** 2022-04-25

**Authors:** Gerhard A. Burger, Bob van de Water, Sylvia E. Le Dévédec, Joost B. Beltman

**Affiliations:** Division of Drug Discovery and Safety, Leiden Academic Centre for Drug Research, Leiden University, Leiden, Netherlands

**Keywords:** cell migration, single-cell analysis, pseudopods, computational modeling, cellular Potts model, breast cancer

## Abstract

The ability of cancer cells to invade neighboring tissue from primary tumors is an important determinant of metastatic behavior. Quantification of cell migration characteristics such as migration speed and persistence helps to understand the requirements for such invasiveness. One factor that may influence invasion is how local tumor cell density shapes cell migration characteristics, which we here investigate with a combined experimental and computational modeling approach. First, we generated and analyzed time-lapse imaging data on two aggressive Triple-Negative Breast Cancer (TNBC) cell lines, HCC38 and Hs578T, during 2D migration assays at various cell densities. HCC38 cells exhibited a counter-intuitive increase in speed and persistence with increasing density, whereas Hs578T did not exhibit such an increase. Moreover, HCC38 cells exhibited strong cluster formation with active pseudopod-driven migration, especially at low densities, whereas Hs578T cells maintained a dispersed positioning. In order to obtain a mechanistic understanding of the density-dependent cell migration characteristics and cluster formation, we developed realistic spatial simulations using a Cellular Potts Model (CPM) with an explicit description of pseudopod dynamics. Model analysis demonstrated that pseudopods exerting a pulling force on the cell and interacting *via* increased adhesion at pseudopod tips could explain the experimentally observed increase in speed and persistence with increasing density in HCC38 cells. Thus, the density-dependent migratory behavior could be an emergent property of single-cell characteristics without the need for additional mechanisms. This implies that pseudopod dynamics and interaction may play a role in the aggressive nature of cancers through mediating dispersal.

## 1 Introduction

Breast Cancer (BC) is the most common cancer in women, and one of the main contributors to cancer mortality (WCRF, 2021). The primary cause of cancer mortality is metastasis, yet, because of its complexity, metastasis remains poorly understood (Suhail et al., 2019; Fares et al., 2020). Migration of cancer cells plays a crucial role in the metastatic cascade, not only for the long-range translocation of cells from the primary tumor to potential metastatic sites but also for the short-range dispersal of cells within the tumor, thus allowing accelerated tumor growth (Waclaw et al., 2015; Gallaher et al., 2019). A detailed understanding of cancer cell migration is essential to obtain insight into cancer progression and metastasis (Stuelten et al., 2018), especially since expression of genes associated with migration is strongly associated with breast cancer survival (Nair et al., 2019).

A complicating factor in studying cancer cell migration is that BC is a highly heterogeneous disease. Two methods that are used to subdivide BCs into clinically-relevant subtypes are gene expression profiling and hormone receptor status (Viale, 2012). PAM50, a 50-gene classifier, divides BC into five intrinsic subtypes: luminal A, luminal B, basal-like, HER2 (human epidermal growth factor receptor 2)-enriched, and normal-like (Perou et al., 2000; Sørlie et al., 2001; Parker et al., 2009). This classification largely corresponds to classification by ER (estrogen receptor), PR (progesterone receptor), and HER2 status. BCs that are negative for these three receptors are called Triple-Negative Breast Cancers (TNBCs). Claudin-low BC, recently redefined as a BC phenotype rather than an intrinsic subtype (Fougner et al., 2020), is characterized by its enrichment for Epithelial-Mesenchymal Transition (EMT) markers and stem cell-like features (Prat et al., 2010).

Different (breast) cancer cells display a stunning variety in migratory strategies, and various methods have been developed to study these strategies *in vitro* (Kramer et al., 2013; Pijuan et al., 2019), *in vivo* (Beerling et al., 2016), and *in silico* (Szabó and Merks, 2013; Wu et al., 2014; Huang et al., 2015; Te Boekhorst et al., 2016). Collective cell migration, where cells migrate in loosely or closely associated clusters, has been extensively studied in morphogenesis (Mayor and Etienne-Manneville, 2016), yet it is also highly relevant during cancer metastasis (Rørth, 2009; Friedl et al., 2012). For example, in recent years the existence of intermediate EMT phenotypes has been increasingly recognized. Such phenotypes are associated with the collective migration of tumor cell clusters (Brabletz et al., 2018), which can have 23–50 fold increased metastatic potential compared to single cells (Aceto et al., 2014). Despite this attention, many open questions remain regarding the mechanisms at play in collective cell migration (Angelini et al., 2011; Vedel et al., 2013). Recently, Jayatilaka et al. (2017) presented experimental evidence that paracrine IL-6/8 signaling amplified by cell density causes fast migration of MDA-MB-231 BC cells. Another approach was taken by Vedel et al. (2013), who studied the 3T3 fibroblast cells at different densities using computational modeling, thereby demonstrating how complex collective migratory behavior can be an emergent property of single-cell migration properties. Thus, computational modeling is an invaluable tool to understand experimentally observed cell migration behavior, as hypothesized underlying mechanisms can be studied both at the single and collective level (Te Boekhorst et al., 2016). Various computational model formalisms for cell migration exist (reviewed by Van Liedekerke et al., 2015; Te Boekhorst et al., 2016; Buttenschön and Edelstein-Keshet, 2020). The Cellular Potts Model (CPM) (Graner and Glazier, 1992; Glazier and Graner, 1993) is widely used for this purpose owing to its explicit incorporation of cell shape, and its flexibility to describe various biomechanical properties. For example, CPM has been used to model T cell migration behavior (Beltman et al., 2007; Ariotti et al., 2012), collective cell migration (Szabó et al., 2010; Kabla, 2012; Czirók et al., 2013; Szabó et al., 2016), chemotactic migration (using a hybrid CPM) (Vroomans et al., 2012), traction forces by cells on 2D substrates (Rens and Edelstein-Keshet, 2019), actin-inspired shape-driven cell migration (Niculescu et al., 2015; Wortel et al., 2021b), enhanced persistence in cooperatively aligning clusters (Debets et al., 2021), and glassy dynamics of cells in confluent tissue (Sadhukhan and Nandi, 2021) (see Szabó and Merks (2013); Sun and Zaman (2017); Buttenschön and Edelstein-Keshet (2020) for more elaborate reviews).

Here we study the migratory behavior of HCC38 and Hs578T, two highly migratory and invasive, claudin-low, basal B TNBC cell lines (Neve et al., 2006; Herschkowitz et al., 2007; Kao et al., 2009; Prat et al., 2013), using time-lapse microscopy and computational modeling with the CPM. To investigate whether these cells exhibit disparate behavior at different cell densities, we plated these cells at various densities and performed 2D cell migration assays using Differential Interference Contrast (DIC) and fluorescence microscopy. In this setting, cell density clearly affected cell migration characteristics such as clustering, speed, and persistence for HCC38 cells, yet not for Hs578T cells. Specifically, at low densities, HCC38 cells formed tight clusters which loosened at high densities; this coincided with increased speed and persistence. We could not reproduce these density effects with published CPM models describing persistent cell migration, yet an extension of a CPM model of pseudopod-driven persistence with a pulling force mediated by pseudopods and increased adhesion at pseudopod tips was sufficient to achieve the experimentally observed speed and persistence increase for HCC38 cells. Thus, pseudopodial dynamics can explain speed and persistence increase with density, provided that the pseudopods of a cell have the ability to affect each other’s extension.

## 2 Results

### 2.1 HCC38 and Hs578T Cell Lines Both Form Streams During *In vitro* Imaging

**FIGURE 1:**
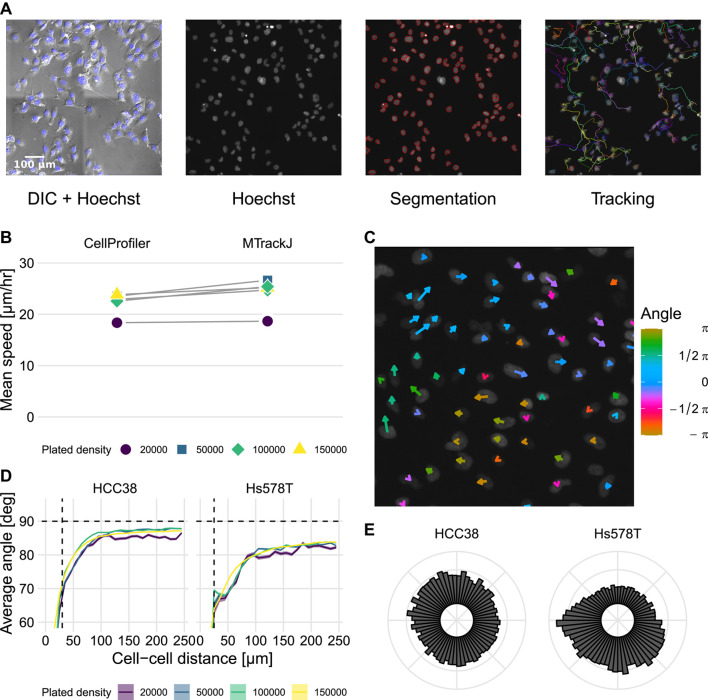
Identification of streams in automatically tracked videos of HCC38 and Hs578T. **(A)** Experimental setup and tracking workflow: nuclei were segmented using Hoechst, after which they were tracked. Images show HCC38 cells at 50,000 cells per well (frame 50 out of 71 frames). **(B)** Measured speed from automated tracking using CellProfiler and from manual tracking using MTrackJ. **(C)** Local migration directions at one time point in Hs578T cells. Size and color indicate instantaneous speed and current direction of migration. Image is a magnification of the top left corner of the bottom-rightmost video in Supplementary Video S1 (Hs578T, 50,000 cells per well, frame 51/71). **(D)** Analysis of migration angles between cell pairs as a function of the shortest distance between their centers of mass, at indicated plated cell densities. Horizontal dashed lines show theoretically expected average angle for random migration, vertical dashed lines show approximate nuclei diameter. **(E)** Polar histogram of migration directions of HCC38 and Hs578T cells. Note that “plated density” in **(B,D)** refers to the number of cells introduced into an entire well.

To investigate the migratory behavior of the TNBC cell lines HCC38 and Hs578T, we plated these lines in triplicate at 4 different cell densities within 24-well plates (20,000, 50,000, 100,000, and 150,000 cells per well). Subsequently, we performed a Random Cell Migration (RCM) assay (van Roosmalen et al., 2011) using DIC and fluorescence microscopy of Hoechst-stained cells, imaged every 11–13 min for approximately 15 h (Supplementary Figure S1 and Supplementary Video S1). To quantify the migratory behavior of cells, we performed automated cell tracking (Figure 1A, see Section 4.3.1 for details) by first segmenting the nuclei using Watershed Masked Clustering (WMC) (Yan and Verbeek, 2012) and then tracking the segmented nuclei in CellProfiler (Carpenter et al., 2006) using overlap tracking. Because of vignetting following stitching of adjacent images (see Figure 1A DIC + Hoechst) and the high densities of cells in some fields of view (Supplementary Figure S1), some segmentation errors still occurred. Since these can affect the quantification of migration characteristics such as cell speed (Beltman et al., 2009), we compared our automated tracking to manually determined tracks in a subset of wells. Analysis of the two methods of tracking revealed that they resulted in similar estimates for cell speed yet that automated tracking led to slightly lower instantaneous cell speeds compared to manual tracking (Figure 1B). This minor difference could be explained by an overestimation of cell speed due to variability in manual center-of-mass determination (Huth et al., 2010). Therefore, and because overall differences between wells were similar for the two tracking approaches, we continued our analysis using automated tracking.

A particularly striking feature that can be appreciated from the time-lapse videos is that Hs578T cells form “streams” (Supplementary Video S1, lower right; clockwise flow in Figure 1C). To quantify this streaming behavior, we analyzed the migration directions of all cells compared to the directions of all other cells. If cells were to migrate randomly, the average angle between two cell migration directions should approach 90° (Beltman et al., 2009). Consistent with the observation of streams within the videos, close-by cells had a lower average angle between their migration directions than remote cells (Figure 1D). This streaming effect was more pronounced for Hs578T cells than for HCC38 cells and occurred at all densities, although visually, it was mainly apparent at high densities. For both cell lines, but especially for Hs578T, the average angle remained below 90° at all densities, which suggests a preferred direction of migration within wells. We confirmed this finding by polar histograms of cell migration direction (Figure 1E), showing that the migration directions of HCC38 cells were approximately uniformly distributed, whereas Hs578T displayed a clear bias in migration direction. However, such large-scale streams could also be due to stage drift (Beltman et al., 2009). Therefore, we took advantage of having two imaged locations per well (technical replicates), for which it would be expected that the polar plots would look highly similar if the streaming effects were due to stage drift. The individual polar plots of two associated well locations frequently exhibited a directional bias for Hs578T cells, yet this bias was typically different between the locations (Supplementary Figure S2A), strongly suggesting that streaming was not due to stage drift. Besides the presence of large-scale streams, both cell lines exhibited strong local streams, as evident from the strong decrease of migration angles for nearby cell movements compared to remote movements (Figure 1D). Moreover, this difference in angle profiles remained present after correction for large-scale streams by subtracting the net overall displacement from each frame (Supplementary Figure S2B). In conclusion, both HCC38 and Hs578T cells formed local streams at all observed cell densities, and especially for Hs578T these streams occurred at a scale beyond the employed image dimensions.

### 2.2 HCC38 Cells Form Dynamic Clusters at Low Densities

**FIGURE 2:**
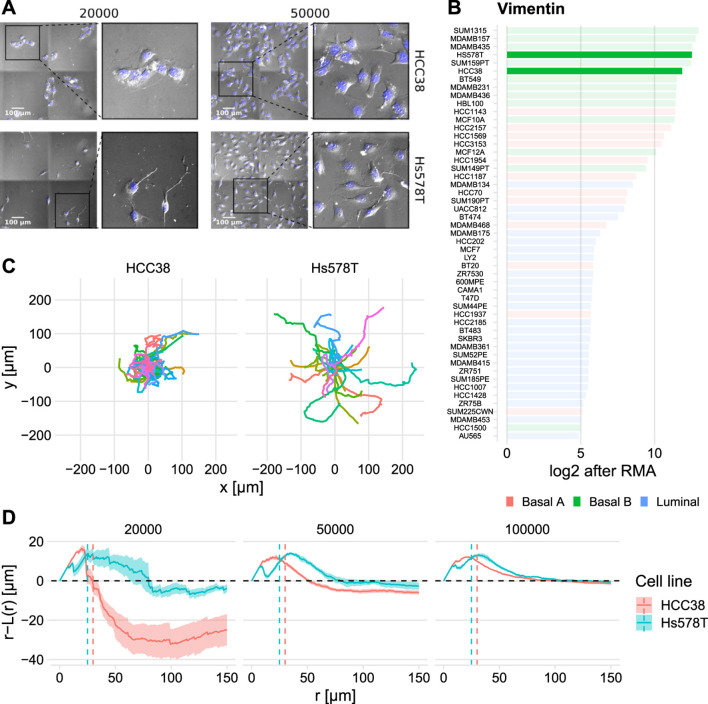
Quantification of observed dynamic clustering of HCC38 cells. **(A)** Still images of HCC38 (top row) and Hs578T (bottom row) at the two lowest densities at t ≈ 10 h (insets show zoom-ins). **(B)** Vimentin expression of a collection of BC cell lines (data from Neve et al., 2006). Values are in log2 after Robust Multi-array Average (RMA). **(C)** Difference in maximum outreach of cells illustrated by normalized track plots (i.e., the starting point is moved to the origin). Data displayed from the same wells as **(A)** left column. **(D)** Spatial statistics visualization of HCC38 and Hs578T using the Ripley L function (see Section 4.5 for details) at t ≈ 10 h. The dashed line *r* − *L*(*r*) = 0 shows the theoretically expected outcome in case of complete spatial randomness, values above and below this line signify dispersion and clustering. The vertical dashed lines denote approximate nuclei diameters.

Visual inspection of images of HCC38 and Hs578T cells (Supplementary Video S1) revealed that at low density HCC38 cells formed clusters (Figure 2A top left), whereas Hs578T cells were less closely apposed to each other, although they may still be in contact *via* extended pseudopods (Figure 2A top left). At high densities (Figure 2A top right), clustering became less dominant for HCC38 yet remained similar for Hs578T. This clustering is surprising since HCC38 is a basal B cell line which are typically considered mesenchymal-like because of their high Vimentin levels (Figure 2B). Consistent with differential clustering between the two cell lines at low density, Hs578T cells traveled further than HCC38 cells, as visible from tracks with starting points normalized to the origin (Figure 2C). Nevertheless, the adhesion presumably driving HCC38 clustering did not completely prevent them from escaping these clusters, a feature that seems to be mediated by pseudopod-driven attachment (Supplementary Video S1).

To quantify clustering, we employed spatial descriptive statistics by means of the Ripley L function (Ripley, 1977). Ripley L allows objective quantification of clustering compared to fully random placement of objects within a region, which is especially useful at high densities where differences in clustering are hard to determine by eye. Specifically, negative values for the quantity *r*—*L*(*r*) (see Section 4.5 for details) imply clustering, whereas positive values imply preferred dispersion. Beyond the diameter of an average nucleus (approximately 25 μm for Hs578T and 30 μm for HCC38), HCC38 cells clearly exhibited clustering, yet this clustering gradually disappeared at increasing densities (Figure 2D, red). Hs578T cells did not cluster but rather exhibited preferred dispersion (Figure 2D, cyan), suggesting that these cells actively stay away from close apposition. Note that the initial increase in Figure 2D shows short-range dispersion for both cell lines, which is caused by volume exclusion (the small dips in this initial bump could be the result of occasional oversegmentation). Over time HCC38 clusters became less compact, as evident from an upward shift in the Ripley-L curves (Supplementary Figure S3). In conclusion, HCC38 cells formed clusters at low densities, whereas this was not the case for Hs578T cells.

### 2.3 HCC38 Cells Exhibit Increasing Instantaneous Speed and Persistence With Increasing Density

**FIGURE 3:**
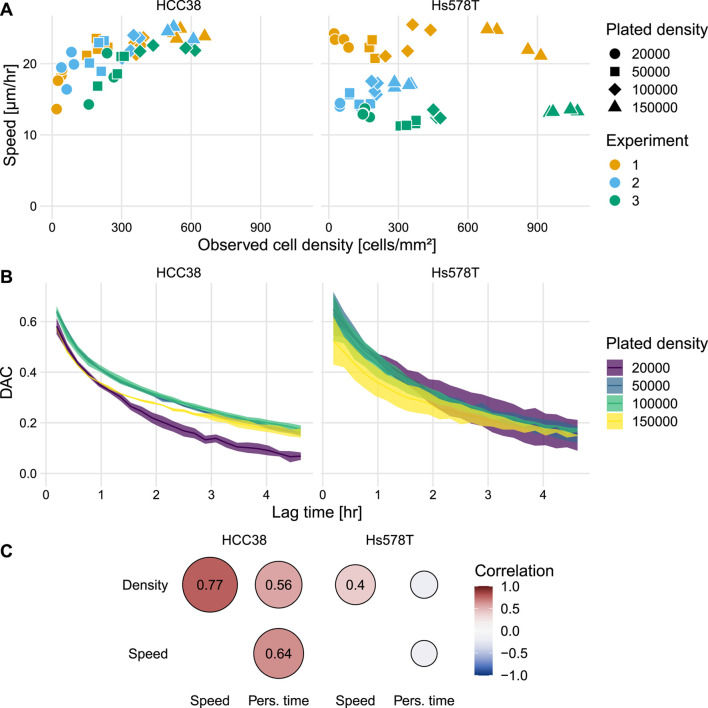
Analysis of the effect of cell density on cell speed and persistence. **(A)** Instantaneous speed for all individual wells as a function of the observed cell density within wells. Colors indicate results for the three separate experiments. **(B)** Mean ± SEM of the DAC as a function of the elapsed time for individual tracks. Note that the data for Hs578T from experiment 2 was excluded from this analysis because the differences in density were very small [see **(A)** and Supplementary Figure S4]. **(C)** Correlogram showing the correlations between the observed cell density, instantaneous speed, and persistence time.

Following our analysis of (collective) migration and clustering, we turned our attention to other aspects of cell migration and whether these depended on cell density and cell type. First, we studied instantaneous speed, for which we investigated the relation with the observed cell densities within wells rather than with the plated densities (Figure 3A), because these might differ due to spatial heterogeneity at different well locations. This speed analysis revealed that HCC38 cells move faster with increasing density (Figure 3A left). In contrast, the speed of Hs578T cells was largely unaffected by cell density (Figure 3A right), i.e., despite substantial experiment-to-experiment variability, there was a similar speed at all densities for each separate experiment.

In addition to cell speed, a short-term measure of migration, we also analyzed the directional persistence of cells, a long-term measure of migration. A commonly used measure of persistence is the confinement ratio [also known as “straightness” (Wortel et al., 2021a) or “meandering index” (Svensson et al., 2018)]. However, since this measure is strongly biased by track duration (Beltman et al., 2009; Gorelik and Gautreau, 2014), it is not suitable for our data which has substantial variation in track duration (Supplementary Figure S5). Instead, we analyzed persistence with Directional Auto Correlation (DAC) (Gorelik and Gautreau, 2014), which represents an unbiased measure of how fast cells lose their direction of migration (see Figure 3A in Gorelik and Gautreau, 2014). Whereas for Hs578T there is no notable difference in the decay of the DAC for different densities, for HCC38 there is a fast decay for the lowest density (Figure 3B). The relation between the DAC and the lag time *τ*_lag_ can be characterized by the following exponential decay function:

$$\phi \exp\left(-\tau_{\text{lag}}/\tau_{p}\right).$$

Here, *τ*_*p*_ is the persistence time and *ϕ* is the persistent fraction, a measure for the fraction of cells that is persistent (Vedel et al., 2013). Calibration of *τ*_*p*_ and *ϕ* for the two cell lines (Supplementary Figure S6) allowed us to study the correlations between cell density, speed, and persistence time. However, it should be noted that the optimal parameter fit did not always describe the decrease in the DAC well (see Supplementary Figure S7), so the resulting parameters should be interpreted cautiously, especially *ϕ*. For Hs578T, an increase in cell density was not associated with an effect on persistence time, but this was the case for HCC38 cells: besides the strong positive correlation between cell density and speed, both cell density and speed also correlated with persistence time (Figure 3C). In conclusion, HCC38 cells strongly increased their speed and persistence for increasing cell densities, whereas the Hs578T cell migration characteristics barely changed for increasing cell densities.

### 2.4 Previous Cellular Potts Migration Models do Not Explain Observed HCC38 Speed-Density Behavior

Our observation that Hs578T cells exhibit dispersion rather than clustering or random positioning in space seems consistent with our findings that speed and persistence do not depend on cell density for this cell line: the cells essentially migrate as solitary cells at all densities. This matches results of an earlier computational model designed for 3T3 fibroblast migration by Vedel et al. (2013), which showed constant speed and persistence time with increased density and a decreased persistent fraction due to a high collision rate. However, for HCC38 cells the relation between the observed clustering and the dependence of cell migration properties on density is less obvious. Therefore, we employed computational modeling to find the minimal requirements to achieve the observed HCC38 density effects. A natural framework to model cell migration is the Cellular Potts Model (CPM), which is a grid-based formalism where multiple grid elements constitute a cell, and membrane elements stochastically extend and retract on the basis of a Hamiltonian. In the base CPM (see Section 4.6 for details), cell motion is driven only by adhesion and cell volume requirements, therefore, cells display Brownian motion, i.e., without any preferred direction and/or persistence (Wortel et al., 2021b). To model persistent cell migration realistically, several extensions to the CPM have been proposed: 1) the “basic persistence model” (see Section 4.6.1.1 for details) in which membrane extensions that move a cell in the same direction as its target direction (derived from previous movement directions) are favored (Beltman et al., 2007; Szabó et al., 2010), and 2) the Act model (see Section 4.6.1.2 for details), which provides cells with persistence by modeling local actin dynamics (Niculescu et al., 2015; Wortel et al., 2021b).

**FIGURE 4:**
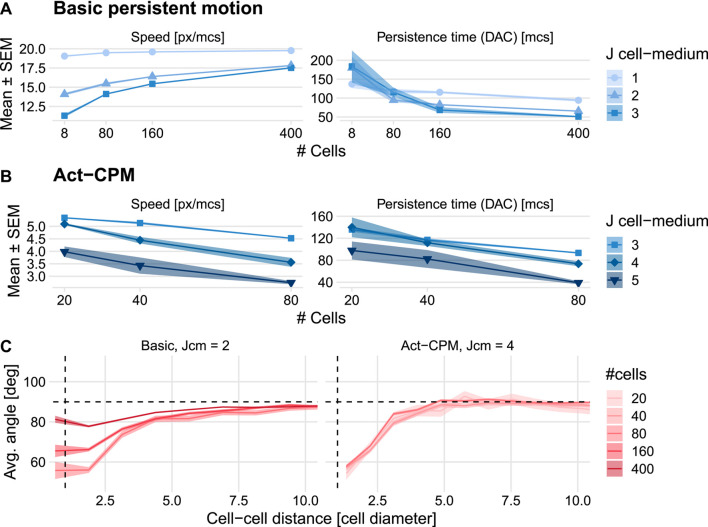
Effect of cell density on cell speed and persistence simulated using existing CPM persistence models. **(A,B)** Instantaneous speed and persistence time as a function of cell density for simulations with the basic persistence mechanism implemented in the Morpheus PersistentMotion plugin **(A)** and the Morpheus Act model implementation **(B)**. Persistence time was in both cases fitted from the DAC in model simulations. **(C)** Stream formation in both models. Horizontal dashed lines denote theoretically expected average angle, vertical dashed lines denote approximate cell diameter.

We explored a wide range of parameter values for both the basic persistence model and the Act model to investigate whether either of these CPM persistence extensions could reproduce the increases in speed and persistence with density observed in the HCC38 cell line. The basic persistence model does exhibit an increase in cell speed for increasing densities, yet there is no corresponding increase in persistence time (Figure 4A). This finding goes against the universal coupling between speed and persistence that has been observed across many cell types in various *in vitro* and *in vivo* settings (Maiuri et al., 2015). Interestingly, the speed increase with density turns into a speed decrease with density when a connectivity constraint (Merks et al., 2006) is added that requires all membrane elements of a cell to remain in touch at all times, i.e., when cell merging is hindered (Supplementary Figure S8 and Supplementary Video S2). This suggests that the increase in speed depends on cells being able to merge and move through each other. The Act-CPM model matches the observed data worse than the basic persistence model, exhibiting a decrease in both speed and persistence with increasing density (Figure 4B and Supplementary Video S3). Analysis of stream formation revealed that both CPM persistence models form local streams where cells align over a maximum of approximately three cell diameters (Figure 4C). In conclusion, although published CPM extensions to describe cellular persistence lead to local streaming, these models are not consistent with the density dependence of HCC38 cell migration.

### 2.5 Pseudopod Dynamics and Increased Pseudopod Interaction can Explain Density-Dependent Migratory Behavior of HCC38 Cells

**FIGURE 5:**
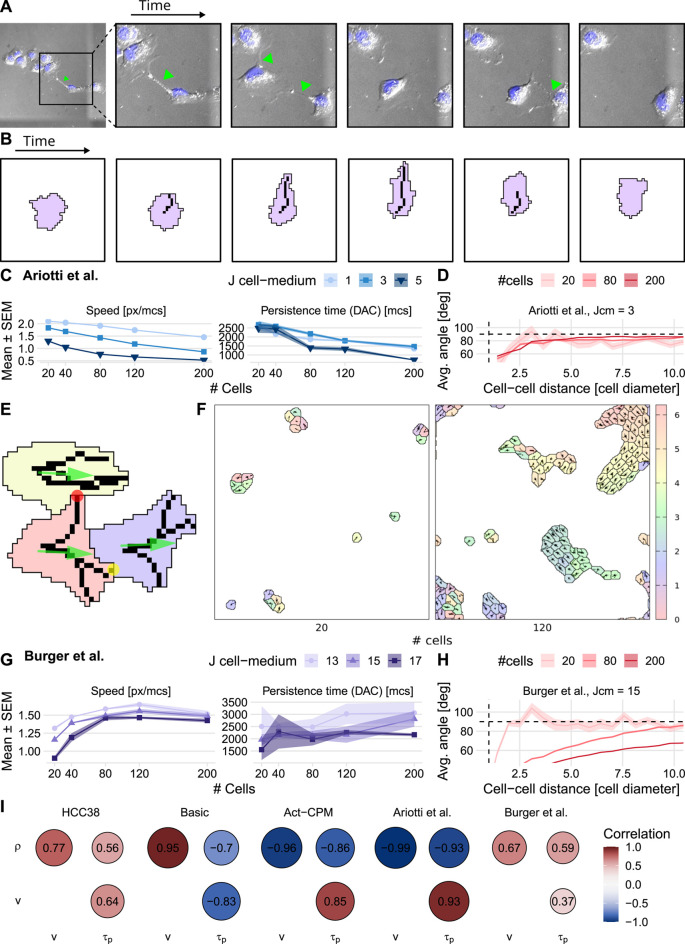
Effect of cell density on cell speed and persistence simulated using pseudopod-driven CPM persistence models. **(A)** Example of HCC38 cells at low density crossing a cluster using pseudopods (green arrows). **(B)** Illustration of how modeling protrusion/retraction of an actin fiber in a cell drives pseudopod-driven motility. **(C)** Results from the base pseudopod-driven model by Ariotti et al. (2012) **(D)** Stream formation in model by Ariotti et al. (2012) **(E)** Mechanisms added in the proposed model: pseudopod tips that are more adhesive (yellow circle), a pulling force in combined direction of the pseudopods (green arrows), and Contact Inhibition of Locomotion-like pseudopod interaction (red circle). **(F)** Snapshot of simulations using the proposed pseudopod-driven persistence model. Densities are comparable to HCC38 plated density of 20,000 and 50,000 (cf. Supplementary Figure S1). **(G)** Speed (left panel) and persistence (right panel) resulting from the proposed pseudopod-driven model. **(H)** Stream formation in the proposed pseudopod-driven model for different simulated cell numbers. **(I)** Correlograms comparing the different persistence models to the experimental correlations (*ρ*: density, *v*: speed, *τ*_*p*_: persistence time). Horizontal dashed lines in **(D)** and **(H)** denote theoretically expected average angle, vertical dashed lines show approximate cell diameter.

Pseudopod formation is essential for persistent cell migration (Bergert et al., 2012; Van Haastert, 2011), and we observed high pseudopodial activity in the experimental videos, which seemed instrumental in HCC38 cells being able to move between clusters (Figure 5A and Supplementary Video S1). Cells simulated with the CPM extensions implementing either basic persistence or the Act model do exhibit a non-roundish shape with small extensions, but these are far shorter than the experimentally observed pseudopods, raising the question of whether the pseudopods might play a role in the observed density effects. Therefore, we implemented our previously developed CPM extension in Morpheus that was used to simulate the migration of dendritic-shaped tissue-resident memory T cells (Ariotti et al., 2012). In this model, cells form dendrite-like protrusions in the form of organized actin bundles that extend and retract within the cell, and that move the cell in the direction of the protrusions (Figure 5B; see Section 4.6.1.3 for details). Although this model could achieve the dynamic clustering observed in HCC38 (Supplementary Video S4), it could still not reproduce the experimentally observed dependence of speed and persistence on density (Figure 5C). Similar to the Act model, the average speed decreased for increasing cell densities, presumably because the fixed dendrites obstruct each other’s extensions, thereby hampering (collective) migration, resulting in limited stream formation (Figure 5D).

In order to test whether increased interaction between cellular pseudopods of a cell would matter for the dependence of migration on cell density, we adapted the modeled behavior of pseudopods in three ways (Figure 5E; see Section 4.6.2 for details): First, we added an adhesive bonus to the pseudopod tips, because close observation of the experimental videos suggested that the pseudopods allow cells to attach to and pull on each other. Second, to further stimulate collective migration in which cells promote rather than hamper each other’s migration, we implemented a type of Contact Inhibition of Locomotion (CIL) (Stramer and Mayor, 2017), where protrusions that are not aligned with the current overall movement direction of a cell and are touching other cells are quickly retracted and repolarized. Third, we let the pseudopods exert a pulling force on the cell as a whole in their combined direction (similar to Vedel et al., 2013). These three mechanisms together result in collective migration of clusters (Figure 5F and Supplementary Video S5). Importantly, our extended pseudopod model could explain the experimentally observed speed and persistence time increase with density for HCC38 cells (Figure 5G, cf. Supplementary Figure S6). The simulated collective migration also goes hand-in-hand with cell alignment over whole clusters, which can be appreciated from the streaming quantification (Figure 5H). Note though that there is a direct dependence of the strength of the streaming on cell density in the simulations, whereas this is not the case in the experimental data (cf. Figure 1D). This cell-density dependence is less pronounced at lower surface energies *J*_cell,med_, for which there is also less long-range alignment (Supplementary Figure S9).

Finally, we investigated the relative importance of the three added mechanisms affecting pseudopod dynamics (pseudopod pulling, pseudopod adhesion, and pseudopod touch behavior, including CIL) for the relation between cell density, speed, and persistence (Figure 5E). When varying the pulling strength and the adhesive tip bonus, we found that pulling strength primarily increases density-dependent persistence (Supplementary Figure S10A and Supplementary Video S6), although with a low adhesive tip bonus cells remain stuck in rotating clusters (Supplementary Figure S10A and Supplementary Video S6, top two rows). Additionally, with low pull strength, individual cells cannot overcome the high surface energy between cell and medium, *J*_cell,med_ (Supplementary Figure S10A and Supplementary Video S6, second column). Hence, with increasing density, fewer cells are stuck, which results in high correlations. In contrast, the effect of pseudopod adhesion is that it promotes dynamic cell behavior inside clusters, without displaying cluster rotation; however, without a pulling force, these clusters barely move collectively (Supplementary Video S6, bottom left), leading to a negative correlation between cell density and persistence (Supplementary Figure S10A, bottom left). A combination of pseudopod pulling and pseudopod adhesion is needed to obtain the dynamic collective migration with a density-dependent speed and persistence time qualitatively matching HCC38 behavior (Supplementary Figure S10A and Supplementary Video S6, bottom right). Varying the touch behavior of pseudopods reveals that this can fine-tune intercellular pseudopod interaction, but that this is not essential for reproducing the observed density-dependent speed and persistence increase; it merely influences the range of surface energies between cells and medium (*J*_cell,med_) for which the CPM simulations exhibit this behavior (Supplementary Figure S10B).

In conclusion, our extended pseudopod model can explain an increase of speed and persistence time with increasing cell density as we observed for HCC38 cells, where other CPM migration models cannot (Figure 5I). In this CPM extension, the presence of CIL promotes these density effects, but pseudopod pulling and adhesion are essential determinants. Thus, pseudopod interaction between cells is an attractive explanation for the HCC38 migration patterns with density.

## 3 Discussion

TNBC is an aggressive subtype of breast cancer for which targeted therapies are just recently showing some promise (McCann et al., 2019). Since migration plays a crucial role in the metastatic cascade, more insight into the mechanisms behind TNBC migratory behavior could help identify potential targets for therapeutic intervention. Here we have used a combination of time-lapse microscopy and computational modeling to unravel the migratory behavior of HCC38 and Hs578T, two highly migratory TNBC cell lines. Both cell lines formed streams in our *in vitro* setup, yet this was most clear from visual inspection in Hs578T cells. HCC38 cells formed dynamic clusters at low density, which became less cohesive at high densities. Furthermore, HCC38 cells exhibited an increase in both speed and persistence time with increasing density. We could not reproduce this density dependence with CPM simulations implementing previously published persistence models, but a pseudopod-driven persistence model with pseudopod-mediated pulling and increased adhesion of pseudopod tips could reproduce the key features of the experimentally observed HCC38 migratory behavior.

Given that HCC38 is a basal B TNBC cell line with very high Vimentin expression (Figure 2B), one would expect that HCC38 is a mesenchymal cell-line (mentioned as such by Hollestelle et al., 2010; Kim et al., 2019). Thus, it was surprising that HCC38 cells strongly clustered, which is typically indicative of an intermediate EMT phenotype (Bocci et al., 2019). A possible explanation is that HCC38 is composed of epithelial and mesenchymal cells at a fixed ratio (as reported by Yamamoto et al., 2017). However, we could not identify subpopulations in our images, nor was this obvious in our single-cell migration analysis. Two indications that HCC38 is, in fact, a hybrid epithelial/mesenchymal cell line are that HCC38 has 1) high P-Cadherin expression (Kao et al., 2009), indicative of an intermediate EMT phenotype (Ribeiro and Paredes, 2014), and 2) high EpCAM (Epithelial Cell Adhesion Marker) expression (Klijn et al., 2015; Koedoot et al., 2021). Especially the increased EpCAM seems relevant because it has been reported to trigger “the formation of dynamic actin-rich protrusions” (Guillemot et al., 2001). Moreover, following EpCAM overexpression cell interactions are reduced to “sporadic contacts, mainly involving filopodia-like structures” (Litvinov et al., 1997), a description that matches our HCC38 observations [Supplementary Video S1, cf. Figure 2 in Winter et al. (2003)]. This suggests EpCAM could play an important role in shaping pseudopodial interactions between HCC38 cells, and thereby in their migration characteristics. Future research should explore the (heterogeneity in) expression of these EMT markers and their relation to the observed pseudopodial dynamics. In addition, the role of potentially density-dependent EMT should also be investigated as, for example, MDCK cells secrete latent TGFβ, a potent EMT inducer, and activate the latent TGFβ in subconfluent conditions (Moyano et al., 2010).

Computational modeling of pseudopod-driven motility is a long-standing challenge (Schindler et al., 2021), and the incorporation of appropriate pseudopod mechanics in our CPM simulations was not straightforward. For example, based on the experimental images, we aimed for long, finger-like extensions; however, for an approximately constant cell area, such long pseudopods easily pull a cell apart in the CPM. One solution could be to use a compartmentalized CPM with a separate nucleus and cytoplasm (Scianna and Preziosi, 2021). However, other model formalisms incorporating physical mechanisms in a spatially implicit manner [e.g., an Agent-Based Model (ABM) as applied in Vedel et al. (2013)] also represent an appropriate way to model pseudopod dynamics and their intercellular interactions.

Our finding that HCC38 cells increase their speed and persistence with increasing cell density is somewhat exceptional. Earlier studies have usually reported cell speed to decrease (Angelini et al., 2011; Guisoni et al., 2018) or stay the same (McCann et al., 2010; Vedel et al., 2013) with increasing density. However, recently it has been shown that in MDA-MB-231, another claudin-low basal B TNBC cell line, paracrine IL-6/8 signaling amplified by cell density does cause faster migration for high than for low densities (Jayatilaka et al., 2017). Other examples of a cell-density-related speed increase include cell motion in endothelial monolayers (Szabó et al., 2010) or confined cell migration (Liu et al., 2015; Szabó et al., 2016). The contexts in which these other experiments were executed are somewhat different compared to our experimental setup, in which the speed increase occurred already at quite a low density (Figure 3A). Nevertheless, it is possible that also in our experimental setting, the observed density effects are (partially) due to a density-dependent nutrient gradient or chemotactic/chemokinetic signal. For future research, we propose experimental exploration of the potential role of such soluble signaling factors to explain increasing cell speed and persistence with cell density. If there indeed is such a role for soluble factors in determining density-dependent migration characteristics, further CPM simulations can assist in distinguishing between chemokinetic and chemotactic effects. Still, we here showed that these effects are not necessary to explain the observed density-dependent migration. This is also confirmed by recent modeling work by Debets et al. (2021), who showed a density-dependent speed and persistence increase for persistently migrating cell clusters of increasing size. In their work, cellular persistence is achieved by implementation of a persistent random walk combined with alignment within clusters that is achieved by explicit neighbor-induced cell polarity updates using a Vicsek model (Debets et al., 2021). Our model is more mechanistic, because both cellular persistence and alignment emerge from the included single-cell pseudopod dynamics.

Based on our simulations, it seems that cells at low density can get “stuck” in their respective clusters (Supplementary Video S5), which is similar to the experimental observations (Supplementary Video S1 top left). At high densities in our simulations, the clusters interact more, thereby avoiding rotating clusters, which causes an increase in persistence and speed. Nevertheless, at high densities, the differences between simulations and experiments become more pronounced; whereas in the experiments the clusters became less cohesive, the simulations exhibit no difference with respect to cohesion (compare Figure 1A HCC38 50,000 with Figure 5F 120). This is also reflected in the streams that form during simulations: Within the large migrating clusters that occur at high cell densities, cells’ migration directions become aligned over large distances (Figure 5H). Lowering the surface energy between the cells and the medium *J*_cell,med_ results in less cohesive clusters and a shorter-range alignment (Supplementary Figure S9). This suggests that cell adhesion might be decreased at high densities compared to low densities, which might also contribute to the high cell speeds at high density (see for example Figure 5G).

In conclusion, in this study we shed light on the influence of cell density on the migratory behavior of two TNBC cell lines, HCC38 and Hs578T. We could reproduce the experimentally observed density-dependent speed increase in HCC38 cells using a pseudopod-driven CPM with pseudopod pulling and increased adhesion at pseudopod tips. A better understanding of the regulatory processes involved in pseudopod formation is urgently needed since they correlate with poor patient survival in multiple cancer types (Jacquemet et al., 2016). Our finding that pseudopod interaction can exacerbate the speed and persistence of cancer cells may be a partial explanation for the aggressive nature of such cancers due to high metastatic potential.

Additionally, together with a previous report that showed how cell density affects the expression of cell-adhesion molecules (Stanley et al., 1995), the data presented here emphasize the need to include appropriate density-related controls in cell-migration assays.

## 4 Materials and Methods

### 4.1 Cell Culture

Twenty-four hours prior to imaging, HCC38 (ATCC Cat# CRL-2314, RRID:CVCL_1267) and Hs578T (ATCC Cat# HTB-126, RRID:CVCL_0332) cells were seeded in complete medium on 24-well glass bottom plates (Sensoplate, Greiner Bio-One, 662892) coated with collagen (rat tail Type I, 10 μg ml^−1^), with the layout as shown in Supplementary Table S1. The seeded densities were 20,000, 50,000, 100,000, and 150,000 cells per well, which, assuming uniform distribution in the well, corresponds to approximately 100, 250, 500, and 750 cells/mm^2^. One hour before imaging, live Hoechst was added to the medium, and just before imaging the medium was refreshed (without additional Hoechst). The experiment was performed in triplicate.

### 4.2 Microscopy

To allow nuclear tracking, cells were incubated for 1 h with Hoechst 33342 https://www.sigmaaldrich.com/NL/en/product/sigma/14533. After incubation, the medium was refreshed, and the plate was directly placed on an automated stage of a Nikon Eclipse TI equipped with a fluorescent lamp and ×20 objective [Plan Apo, Air, numerical aperture (NA) 0.75, working distance (WD) 1.0], a Perfect Focus System (PFS) and a temperature- and CO_2_-controlled imaging chamber (custom design). Two positions per well were imaged using both fluorescence and DIC microscopy. The plates were imaged at 999 × 999 px (experiment 1) or 948 × 948 px (experiment 2 and 3) using a stitch of 2 × 2 positions with a pixel size of 0.79 µm. The imaging was repeated every 11 min (experiment 1) or 13 min (experiment 2 and 3) for 15 h. Images are available at https://dx.doi.org/10.5281/zenodo.5607734 (Le Dévédec, 2021).

Upon visual inspection of the microscopic images, we noted that for the third experiment of the HCC38 150,000 condition, cells were dying; therefore, we excluded these wells from further analysis.

### 4.3 Image Processing

The imaging processing and analysis consisted of multiple steps. Initially, proprietary Nikon ND2 image files were converted to the Tagged Image File Format (TIFF) using NIS-Elements (NIS-Elements, RRID:SCR_014329).

#### 4.3.1 Automated Tracking

Subsequently, the resulting TIFFs were processed in a CellProfiler pipeline (CellProfiler Image Analysis Software, V2.1.1, RRID:SCR_007358) (Carpenter et al., 2006), containing the following steps:

• Cropping: Following stitching of the images, they contained zero-intensity patches at the edges as a result of (mis)alignment. To avoid problems with segmentation and edge detection later in the pipeline, we cropped the images by 2 pixels at the edges.
• Segmentation: The cropped images were segmented using the WMC approach (Yan and Verbeek, 2012). See Supplementary Table S2 for the utilized parameters.
• Object identification: We converted the connected components in the segmented images into objects. The resulting objects were filtered on size; we only retained objects with a diameter between 10 px (8 μm) and 40 px (32 µm) for Hs578T, or 50 px (40 µm) for HCC38. Additionally, we discarded objects touching the image border to prevent inaccurate center-of-mass calculation.
• Tracking: We tracked the remaining objects using the *Overlap* tracking method with a maximal pixel distance of 30.

#### 4.3.2 Manual Tracking

To compare our automated tracking to manual tracking, we used MTrackJ (Meijering et al., 2012) in Fiji (Fiji, RRID:SCR_002285) (Schindelin et al., 2012; Rueden et al., 2017) to manually track a representative subset of the wells by clicking the center of mass of each cell in each frame. Although manual tracking is considered the gold standard for tracking (Cordelières et al., 2013), variability in center-of-mass determination (e.g., due to operator fatigue) can cause an overestimation of the actual cell speed (Huth et al., 2010).

#### 4.3.3 Nucleus Diameter Calculation

Because the cells show high pseudopodal activity, the cell diameters are difficult to estimate. Instead, we estimated the nucleus diameters, which are 30 and 25 µm for HCC38 and Hs578T. Using the EBImage R package (Pau et al., 2010), we first applied an adaptive threshold on the Hoechst signal, followed by watershed transformation and object feature analysis. Nucleus diameters were estimated as two times the average nuclei radius reported by EBImage, rounded up. These nucleus diameters serve as an approximation for the nearest possible distance between cells.

### 4.4 Track Analysis

Tracking data from CellProfiler was imported into R using an in-house developed script (Wink and Burger, 2021; Wink, 2015, Ch. 7) and by fixing track identifiers with the CPTrackR package (Burger et al., 2021a). MTrackJ data were imported using the mdftracks package (Burger, 2021).

Analysis in R (R Project for Statistical Computing, RRID:SCR_001905) (R Core Team, 2018) was performed with RStudio (RStudio, RRID:SCR_000432) (RStudio Team, 2016) and with the packages celltrackR (Wortel et al., 2021a), spatstat (Baddeley et al., 2015), and tidyverse (Wickham, 2017) packages.

#### 4.4.1 Directional Autocorrelation

The Directional Auto Correlation (DAC) of all cells was computed by

$$\text{DAC}\left(\tau \right)=\left\langle e_{j\Delta t}^{i}\cdot e_{\left(j+n\right)\Delta t}^{i}\right\rangle ,$$

where $e_{j\Delta t}^{i}$ denotes the normalized direction of motion of cell *i* at time *j*Δ*t*, and the angle brackets denote averaging over all cells *i* and all times *j*Δ*t* and (*j* + *n*)Δ*t*, where Δ*t* is the sampling time, of which the lag time *τ* = *n*Δ*t* is a multiple. We computed the DAC in R using the overallNormDot function, which we contributed to the celltrackR package (Wortel et al., 2021a).

After removing DAC (0), which is by definition equal to unity, we fitted the exponential decay function

$$\phi e^{-\tau /\tau_{p}},$$

which gives an estimate for the weight factor *ϕ* (can be interpreted as the fraction of cells that is persistent) and persistence time *τ*_*p*_ (Vedel et al., 2013). Since the estimates for *ϕ* resulting from parameter calibration were not always reliable (see Supplementary Figure S7), we focused on *τ*_*p*_ in our further analysis.

#### 4.4.2 Correlograms

Averaged correlation values for the experimental correlograms were computed using the Fisher transformation. First, the Pearson correlation *r* for each experiment (biological replicate) was converted into a Fisher’s *z*:

$$z=\frac{1}{2}\ln\left(\frac{1+r}{1-r}\right)=\text{artanh}\left(r\right),$$

where artanh is the inverse hyperbolic tangent. Then *z* can be averaged and converted back with

$$r=\frac{\exp\left(2z\right)-1}{\exp\left(2z\right)+1}=\text{tanh}\left(z\right),$$

where tanh is the hyperbolic tangent (Corey et al., 1998).

### 4.5 Clustering Analysis With Ripley K

To analyze spatial clustering, we used the common transformation on Ripley K (Ripley, 1977) defined as

$$L\left(r\right)=\sqrt{\frac{K\left(r\right)}{\pi }},$$

and provided in the spatstat R package (Baddeley et al., 2015). We subsequently visualized *r*—*L*(*r*) as a function of *r* such that in case of complete spatial randomness

$$L\left(r\right)=r\;\Rightarrow \;r-L\left(r\right)=0,$$

which allows to determine whether clustering (*r*—*L*(*r*) < 0) or dispersion (*r*—*L*(*r*) > 0) occurs.

### 4.6 Cellular Potts Modeling

In the CPM, cells are defined as a collection of lattice sites $\vec{v}\in Z^{n}$ with the same cell identifier *σ*. Each cell also has an associated cell type *τ*(*σ*). At sites forming the cell boundaries (referred to as “membrane elements” below), there is a cell-type-dependent surface energy $J_{\tau_{1},\tau_{2}}$ . A simulation consists of a sequence of Monte Carlo Steps (MCS), during which cells attempt to extend membrane elements that would modify the cell identifier of a lattice site $\sigma \left(\vec{v}\right)$ into the identifier of one of the neighboring lattice sites $\sigma \left({\vec{v}}_{n}\right)$ in the 2D Moore neighborhood without the central square (i.e., the 8 sites of the first- and second-order neighbors in 2D). The probability that such an extension is accepted depends on the change in the Hamiltonian:

$$H=\underset{\vec{v},{\vec{v}}_{n}}{\sum }J_{\tau \left(\sigma \left(\vec{v}\right)\right),\tau \left(\sigma \left({\vec{v}}_{n}\right)\right)}\left(1-\delta_{\sigma \left(\vec{v}\right)\sigma \left({\vec{v}}_{n}\right)}\right)+\lambda_{V_{\tau \left(\sigma \right)}}\underset{\sigma }{\sum }{\left(v\left(\sigma \right)-V_{\tau \left(\sigma \right)}\right)}^{2},$$

where the first term is the sum of the surface energies over all $\vec{v},{\vec{v}}_{n}$ neighbor pairs, and the second term is the elastic area constraint which keeps cells within a range of biologically appropriate sizes. Furthermore, *δ* is the Kronecker delta, $\lambda_{V_{\tau }}$ is the elastic constant for the area of cell type *τ*, *V*_*τ*_ is the target area of cell type *τ*, and *v*(*σ*) is the actual area of cell *σ*.

The probability *p* that an extension is accepted depends on the change in the Hamiltonian Δ*H* as follows:

$$p=\left\{\begin{array}{cc} 1,\; & \text{for }\Delta H\leq 0\\ e^{\frac{-\Delta H}{T}},\; & \text{for }\Delta H>0 \end{array}\right.,$$

where *T* is the temperature (Graner and Glazier, 1992; Glazier and Graner, 1993). We used Morpheus (RRID:SCR_014975, Starruß et al., 2014) for the CPM implementation.

#### 4.6.1 Existing Persistence Models

##### 4.6.1.1 Basic Persistence

In the basic persistence model implemented in the PersistentMotion plugin in Morpheus, cells have a target direction $\vec{t}$ based on previous movements which is updated continuously according to

$${\vec{t}}_{\text{new}}=\left(1-\text{dr}\right){\vec{t}}_{\text{old}}+\text{dr}\Delta \vec{x}/|\Delta \vec{x}|,$$

where dr = min (1/dt, 1) is the decay rate, with decay time dt in MCS, and $\Delta \vec{x}={\vec{x}}_{\text{new}}-{\vec{x}}_{\text{old}}$ is the shift of the cell centroid in the previous MCS. For a proposed copy attempt $\sigma \left(\vec{v}\right)\to \sigma \left({\vec{v}}_{n}\right)$ in update direction $\vec{s}$ , the additional change in Hamiltonian *H* due to persistence is computed as:

$$\Delta H=\underset{\sigma \in S}{\sum }-\lambda_{P}\;v\left(\sigma \right)\left({\vec{s}}_{\sigma }\cdot {\vec{t}}_{\sigma }\right),$$

where $S=\left\{\sigma \left(\vec{v}\right),\sigma \left({\vec{v}}_{n}\right)\right\}$ is the set of involved cells, *λ*_*P*_ the strength of persistence, and *v*(*σ*) the cell area. Note that the operator ⋅ is the dot product. Other implementations of a basic persistence mechanism have also been proposed (e.g., in Beltman et al. (2007); Szabó et al. (2010); Guisoni et al. (2018)), which do not include cell area. An advantage of including cell area in the equation is that it may contribute to a realistic description of inertia. However, in scenarios with a single cell type and only limited variability in cell area over time (as is the case in our simulations), its inclusion represents just a scaling factor on the persistence strength *λ*_*P*_ and is thus expected to have a negligible effect on cell migration characteristics. For details on the parameters used for the basic persistence model, see Supplementary Table S3.

##### 4.6.1.2 Act-CPM

In the Act-CPM model, persistence is achieved by recording each lattice site’s “actin activity,” which depends on the MCS elapsed since its most recent protrusive activity. Upon a successful copy attempt, the target lattice site is assigned the maximum activity value (Max_act_), which decreases every MCS until it reaches zero. By making a copy attempt from an active site into a less active site more favorable, a local positive-feedback mechanism is created, which causes persistent motion. For a proposed copy attempt $\sigma \left(\vec{v}\right)\to \sigma \left({\vec{v}}_{n}\right)$ the additional change in Hamiltonian *H* is computed as

$$\Delta H=-\frac{\lambda_{\text{Act}}}{{\text{Max}}_{\text{Act}}}\left({\text{GM}}_{\text{Act}}\left(\vec{v}\right)-{\text{GM}}_{\text{Act}}\left({\vec{v}}_{n}\right)\right),$$

where ${\text{GM}}_{\text{Act}}\left(\vec{v}\right)$ is the geometric mean of all activities of lattice sites in the Moore neighborhood of $\vec{v}$ which share the same cell identifier $\sigma \left(\vec{v}\right)$ (Niculescu et al., 2015; Wortel et al., 2021b).

For this study, we used the Act-CPM model provided in Morpheus; for details on the parameters used, see Supplementary Table S4.

##### 4.6.1.3 Pseudopod Model Ariotti et al.

In the pseudopod model by Ariotti et al. (2012), pseudopod dynamics are realized by extending and retracting explicitly described actin fibers using a finite state machine (Supplementary Figure S11):

• A pseudopod starts in the INIT state in which its first actin filament is added at the cell center-of-mass (rounded to the nearest pixel location). This addition is only accepted if it is at a location where a cell’s pixel indeed resides. Moreover, a growth direction is drawn from a von Mises distribution centered around the current movement direction of the cell with concentration *κ*_init_ (parameter init-dir-strength).
• When initialized, the pseudopod enters the GROWING state. During each MCS, the actin fiber is extended with probability *p*_ext_ = 0.3; a position for a new actin filament is determined by drawing a direction from a von Mises distribution centered around its current growth direction with concentration *κ*_cont_ (parameter cont-dir-strength). If the new position resulting from an extension in this direction is part of the current cell pixels, the actin filament is added to the pseudopod’s actin bundle, and its growth direction is updated to the direction in which the pseudopod was extended during this MCS.
• When the maximum growth time is reached (max-growth-time) or if no extension has happened for 20 MCS, cells enter the RETRACTING state. In each MCS, the actin fiber is retracted with probability *p*_retr_ = 0.3. There are multiple retraction methods (parameter retraction-mode): 1) backward, where actin fibers are removed from the pseudopod tip, 2) forward, where actin fibers are removed from the origin of the pseudopod, resulting in “treadmilling” (Marée et al., 2007).
• When the actin fiber is completely retracted, the pseudopod enters the INACTIVE state. This state can be used to limit pseudopod activity; every MCS, the cell is moved to the INIT state with probability 1/time−between−extensions .

To couple this finite state machine description of actin fibers to the behavior of CPM pixels, cell growth directly next to actin fiber is promoted, and cell shrinking around actin is prevented. Specifically, for a proposed copy attempt $\sigma \left(\vec{v}\right)\to \sigma \left({\vec{v}}_{n}\right)$ , presence of a site with actin fiber ${\vec{v}}_{a}$ in the 2D Moore neighborhood (including the central pixel itself) of $\vec{v}$ and $\sigma \left({\vec{v}}_{a}\right)=\sigma \left({\vec{v}}_{n}\right)$ , leads to an additional change in the Hamiltonian ΔH=−neighboring−actin−bonus . To prevent cell shrinking around actin, for a proposed copy attempt $\sigma \left(\vec{v}\right)\to \sigma \left({\vec{v}}_{n}\right)$ , presence of a site with actin fiber ${\vec{v}}_{a}$ in the 2D Moore neighborhood (including the central pixel itself) of $\vec{v}$ and $\sigma \left({\vec{v}}_{a}\right)=\sigma \left(\vec{v}\right)$ and $\sigma \left({\vec{v}}_{a}\right)\neq \sigma \left({\vec{v}}_{n}\right)$ , leads to blocking of this attempt by an additional change in Hamiltonian Δ*H* = *∞*, such that the acceptance probability *p* → 0. See Figure 5B for an example of this pseudopod-driven motility with a single pseudopod.

#### 4.6.2 Proposed Model

To obtain realistic pseudopod-driven persistence that matches our experimental observations, we adapted the model by Ariotti et al. (2012) described in Section 4.6.1.3. We implemented this model as a Morpheus plugin [thus also including the version previously published in Ariotti et al. (2012)] and adapted it in several ways to implement different processes involved in the pseudopod dynamics:

• In HCC38 cells *in vitro*, we observed a “stickiness” of pseudopods (Supplementary Video S1). To mimic this effect, we added an adhesion bonus *E*_tip-bonus_ (tip-bonus) to the *in silico* pseudopod tips. The bonus is applied when for a proposed copy attempt $\sigma \left(\vec{v}\right)\to \sigma \left({\vec{v}}_{n}\right)$ , ${\vec{v}}_{n}$ is in the 2D Moore neighborhood of another cell $\sigma_{o}\neq \sigma \left({\vec{v}}_{n}\right)$ , and ${\vec{v}}_{n}$ is within *r*_max_ (max-distance-for-tip-bonus) of one of its own pseudopod tips. Moreover, when this position is also within *r*_max_ of a pseudopod tip from neighboring cell *σ*_*o*_, this bonus is doubled. Thus:

$$\Delta H=\underset{\sigma \in S}{\sum }\left\{\begin{array}{cc} 0,\; & \text{if }r_{\sigma }>r_{\text{max}}\wedge r_{\sigma_{o}}>r_{\text{max}},\\ -E_{\text{tip}-\text{bonus}},\; & \text{if }r_{\sigma }\leq r_{\text{max}}⊻r_{\sigma_{o}}\leq r_{\text{max}},\\ -2E_{\text{tip}-\text{bonus}},\; & \text{if }r_{\sigma }\leq r_{\text{max}}\wedge r_{\sigma_{o}}\leq r_{\text{max}}, \end{array}\right.$$

where $S=\left\{\sigma \left(\vec{v}\right),\sigma \left({\vec{v}}_{n}\right)\right\}$ is the set of involved cells in the copy attempt, *r*_*σ*_ and $r_{\sigma_{o}}$ are the minimum distances between $\vec{v}$ and one of the pseudopods of *σ* and *σ*_*o*_ respectively. Note that this tip adhesion bonus applies to cells that would grow because of the considered expansion. However, if such an expansion promoted by the tip bonus would also lead to shrinkage of another cell, the tip bonus becomes a tip penalty for the second cell involved. Thus, the net Δ*H* would be zero, preventing pseudopods from “poking” into other cells.

• To increase the persistence of the cells, we implemented a pseudopod pulling effect, similar to an effect simulated in Vedel et al. (2013). Given a copy attempt $\sigma \left(\vec{v}\right)\to \sigma \left({\vec{v}}_{n}\right)$ in update direction $\vec{s}$ , the change in Hamiltonian is computed as

$$\Delta H=\underset{\sigma \in S}{\sum }-F\left({\vec{s}}_{\sigma }\cdot {\vec{f}}_{\sigma }\right)/v\left(\sigma \right),$$

where $S=\left\{\sigma \left(\vec{v}\right),\sigma \left({\vec{v}}_{n}\right)\right\}$ is the set of involved cells, *F* the pulling force (pull-strength), ${\vec{f}}_{\sigma }$ the vector sum of all pseudopod vectors (i.e., the vectors from each pseudopod’s origin to its tip), and *v*(*σ*) the cell area (note that here *σ* indicates the cell from which a pixel copy into a neighboring pixel is considered). The net force is divided by cell area because it should be more difficult to accelerate a large (heavy) cell than a small cell. Nevertheless, since we model only one cell type with cells of relatively constant area over time, this is likely to have very only a minor effect.

• To increase the alignment of neighboring cells, we implemented a “touch” strategy inspired by the phenomenon Contact Inhibition of Locomotion (CIL). Specifically, when growth of the actin fiber (see Section 4.6.1.3) is attempted into a neighboring cell, that pseudopod is considered to be “touching.” The actin fiber growth attempt is rejected, after which the following behaviors can happen depending on the touch-behavior parameter:
  • nothing: the simulation continues as before (this is the behavior in the original Ariotti model (Ariotti et al., 2012), which does not consider “touching” as a special event).
  • retract: pseudopod is set to RETRACTING state, allowing eventual reformation of a new pseudopod in a novel direction.
  • attach: to mimic cells latching onto each other with their pseudopods, we introduced a TOUCHING state, where a pseudopod is neither growing nor retracting. Every MCS, there is a probability *p*_touch_retr_ to enter the RETRACTING state. Thus, on average, this introduces a delay before pseudopod retraction occurs upon touching.
  • poof-dir: when a pseudopod touches a neighboring cell laterally (i.e., when cos *α* < 0.85, with *α* the angle between the overall pseudopod direction (vector from origin to tip) and the current movement direction of the cell), the pseudopod is instantly retracted. This implies that the RETRACTING state is omitted and that a new pseudopod can be initialized in a novel direction.

Our final model to represent the behavior of HCC38 cells employs the poof-dir touch strategy, yet we also compare it with the other touch behaviors.

For details on the parameters used, see Supplementary Tables S5, S6. A description of all pseudopod parameters can be found in Supplementary Table S7. The code for the Morpheus plugin is available at https://doi.org/10.5281/zenodo.5484491 (Burger and Beltman, 2021) in the files morpheus/plugins/miscellaneous/gab_pseudopodia.cpp and morpheus/plugins/miscellaneous/pseudopod.cpp. An example Morpheus model using the plugin is available in the file Examples/Miscellaneous/Pseudopodia.xml

#### 4.6.3 Choice of Simulation Parameters

To efficiently explore the parameter space, we used the Python Programming Language (RRID:SCR_008394) in Jupyter Notebook (RRID:SCR_018315) (Kluyver et al., 2016), and FitMultiCell (Alamoudi et al., 2019) [based on pyABC (Schälte et al., 2021) and Morpheus (Starruß et al., 2014)]. Based on this extensive exploration, we selected representative parameter sets that qualitatively matched (parts of) the experimental data. The simulated number of cells was similar to the number of cells observed in experiments (Supplementary Figure S4) and were initialized as randomly placed single pixels on the lattice that quickly grew to values close to their target areas. Rather than using the same pixel dimensions as in the experiments (∼1000 × 1000 px), we used a simulation lattice size of 400 × 400 px for the Ariotti model and our proposed model, equivalent to a CPM pixel size of ∼2 µm. This was done partly to achieve a reasonable run time of individual simulations (∼45 min on an Intel Xeon E5-2660 v3) and to get realistic cell/pseudopod proportions. We used our segmented experimental images to determine the cell target area in our simulations (250 px, compare Figure 1A, HCC38, and Figure 5F), corresponding to a cell diameter of ∼20 µm. All simulations ran for 20,000 MCS. Note that we did not make an explicit choice for the amount of real time that 1 MCS represents, because this would mean that for every parameter change, a new choice would be required to obtain realistic speeds. Rather, migration characteristics were qualitatively compared to experiments by using MCS as a time unit for simulation data.

#### 4.6.4 Simulation Measurements

We saved the cell positions from the simulations every MCS, and after discarding of the first 1,000 MCS to allow for equilibration, we analyzed them in the same way as the experimental cell tracks, except for instantaneous speed, which was estimated based on 50 MCS subtracks.

## Data Availability

The original contributions presented in the study are publicly available. The generated imaging data can be found here: https://doi.org/10.5281/zenodo.5607734 (Le Dévédec, 2021). The computational model can be found here: https://doi.org/10.5281/zenodo.5484491 (Burger and Beltman, 2021).

## Abbreviations

ABM, Agent-Based Model; BC, Breast Cancer; CIL, Contact Inhibition of Locomotion; CPM, Cellular Potts Model; DAC, Directional Auto Correlation; DIC, Differential Interference Contrast; EMT, Epithelial-Mesenchymal Transition; EpCAM, Epithelial Cell Adhesion Marker; ER, estrogen receptor; HER2, human epidermal growth factor receptor 2; MCS, Monte Carlo Step; PR, progesterone receptor; RCM, Random Cell Migration; RMA, Robust Multi-array Average; TNBC, Triple-Negative Breast Cancer; WMC, Watershed Masked Clustering.
