# Supplementary material for: Density-Dependent Migration Characteristics of Cancer Cells Driven by Pseudopod Interaction
Source: Front Cell Dev Biol. 2022 Apr 25;10:854721. doi: 10.3389/fcell.2022.854721 (PMC9084912; doi:10.3389/fcell.2022.854721)
Supplement: Supplementary file 2 [file DataSheet1.pdf]

Supplementary Material

1 SUPPLEMENTARY TABLES AND FIGURES

1.1 Tables

	HCC38		Hs578T		
	1	2	3	4	density
A	1,2	15,16	17,18	31,32	20000
B	3,4	13,14	19,20	29,30	50000
C	5,6	11,12	21,22	27,28	100000
D	7,8	9,10	23,24	25,26	150000

Table S1. Plate layout for the random cell migration assays, the numbers denote the imaging order. There are two wells per condition, and two positions (technical replicates) per well. Wells were imaged by column in a zig-zag pattern.

	HCC38	Hs578T
Gaussian filter size	2	1
Rolling ball size	200	
Use parabolic kernel	No	
Noise	15	12
Low seed	15	
High seed	20	
Low bound	0.4	
High bound	0.7	
Use intensity equalize	No	

Table S2. Settings for the WMC plugin in CellProfiler (see Yan and Verbeek (2012) for further details).

Parameter	Value	Description
A	$200 \times 200\,\text{px}$	Simulation lattice size
$J_{\tau,\tau'}$	$J_{\text{cell,cell}} = 1$	Surface energies between cell types
V_τ	$V_{\text{cell}} = 50\,\text{px}$	Target volume of cell type
λ_{V_τ}	$\lambda_{V_{\text{cell}}} = 1$	Elastic constant for volume
P_τ	$P_{\text{cell}} = 10\,\text{mcs}$	Persistence decay time for cell type
λ_{P_τ}	$\lambda_{P_{\text{cell}}} = 1$	Elastic constant for persistence

Table S3. Parameters for the Morpheus PersistentMotion model used to generate Fig. 4A.

Parameter	Value	Description
A	$200 \times 200\,\text{px}$	Simulation lattice size
$J_{\tau,\tau'}$	$J_{\text{cell,cell}} = 1$	Surface energies between cell types
V_τ	$V_{\text{cell}} = 100\,\text{px}$	Target volume of cell type
λ_{V_τ}	$\lambda_{V_{\text{cell}}} = 1$	Elastic constant for volume
S_τ	$S_{\text{cell}} = 0.9$	Target asphericity (surface constraint) of cell type
λ_{S_τ}	$\lambda_{S_{\text{cell}}} = 0.5$	Elastic constant for asphericity
Max_{Act}	50	Maximum actin activity value
λ_{Act}	10	Maximum contribution of Act model to Hamiltonian

Table S4. Parameters for the Morpheus Act-CPM plugin model used to generate Fig. 4B.

Parameter	Value	Description
A	$400 \times 400\,\text{px}$	Simulation lattice size
$J_{\tau,\tau'}$	$J_{\text{cell,cell}} = 1$	Surface energies between cell types
V_τ	$V_{\text{cell}} = 250\,\text{px}$	Target volume of cell type
λ_{V_τ}	$\lambda_{V_{\text{cell}}} = 1$	Elastic constant for volume
S_τ	$S_{\text{cell}} = 0.9$	Target asphericity of cell type
λ_{S_τ}	$\lambda_{S_{\text{cell}}} = 0.5$	Elastic constant for asphericity

Table S5. Shared parameters for the models used to generate Figs. 5C and 5G.

Parameter	Ariotti	Burger
`max-growth-time`	20	
`max-pseudopods`	3	
`time-between-extensions`	1	
`tip-bonus`	0	30
`max-distance-for-tip-bonus`	n/a	5
`neighboring-actin-bonus`	8	
`init-dir-strength`	8	
`cont-dir-strength`	16	
`retraction-mode`	`forward`	
`touch-behavior`	`nothing`	`poof-dir`
`pull`	false	true
`pull-strength`	n/a	50

Table S6. Parameters for Pseudopodia plugin used together with Table S5 to generate Figs. 5C and 5G. See Table S7 for a description of these parameters.

Parameter	Description
`field`	Morpheus field to keep track of the actin cytoskeleton
`moving-direction`	The (buffered) moving direction of the cell (used to create new pseudopodia in that approximate direction)
`max-growth-time`	Max number of MCS pseudopods grow
`max-pseudopods`	Max number of pseudopods per cell
`time-between-extensions`	Refractory period for pseudopod extension
**`tip-bonus`**	**Adhesion bonus for the pseudopod tip applied to the Hamiltonian.**
**`max-distance-for-tip-bonus`**	**The maximum distance at which surrounding pixels are considered part of the pseudopod tip**
`neighboring-actin-bonus`	Hamiltonian bonus to stimulate growth directly next to actin cytoskeleton
`init-dir-strength`	κ for von Mises distribution to bias pseudopod formation in `moving-direction`
`cont-dir-strength`	Same as `init-dir-strength`, except to bias pseudopod growth direction
`retraction-mode`	• `backward`: pseudopod is retracted backwards<br>• `forward`: pseudopod is "retracted" forwards<br>• `in-moving-direction`: forwards if aligned with `moving-direction`, backwards otherwise.
**`touch-behavior`**	**If pseudopods touch another cell...**<br>• **`nothing`**<br>• **`retract`: retract with `retraction-mode`**<br>• **`attach`: stop pseudopod growth phase and begin delayed retraction phase**<br>• **`poof-dir`: if touching laterally, pseudopod retracts instantaneously**
**`pull`**	**Turn 'pulling' on/off**
**`pull-strength`**	**Hamiltonian bonus if update moves cell in the combined direction of the pseudopods**

Table S7. Parameter description for the developed Morpheus Pseudopodia plugin. **Bold parameters** are parameters introduced specifically for this study.

1.2 Figures

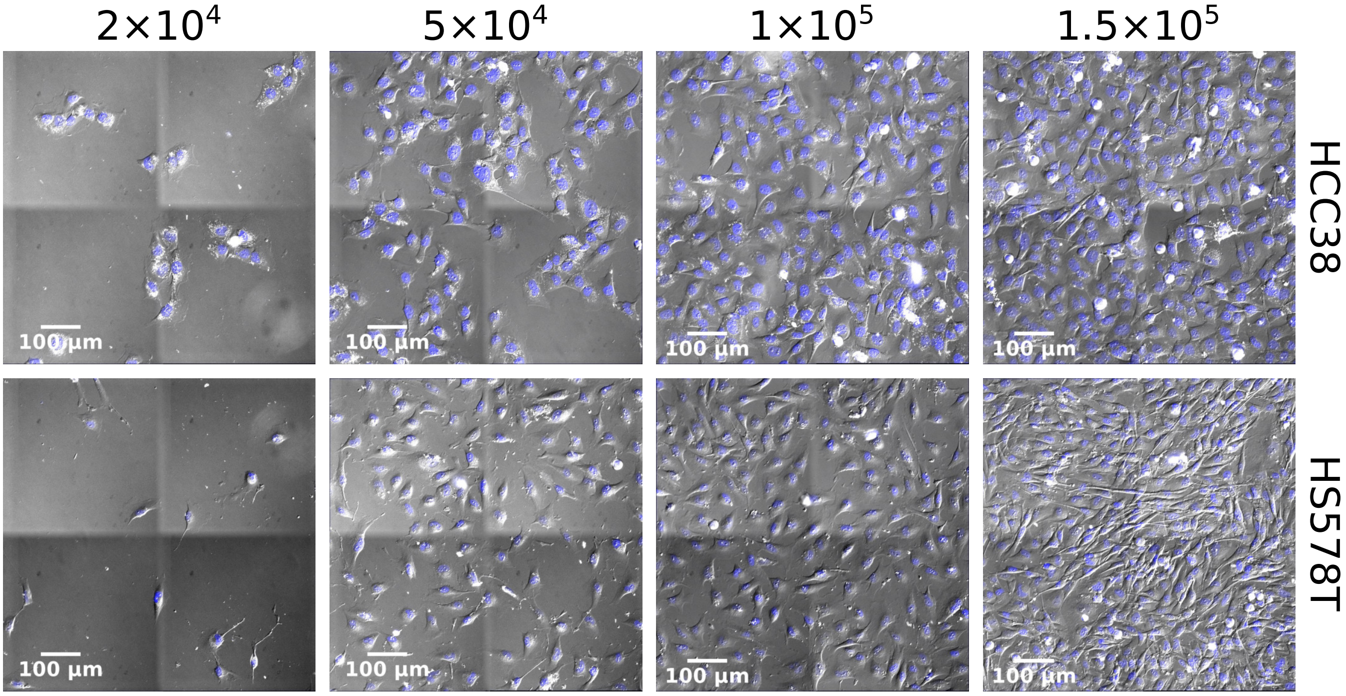

Figure S1. Overview of all experimental plated densities for HCC38 and Hs578T cell lines. See Vid. S1 for the corresponding videos.

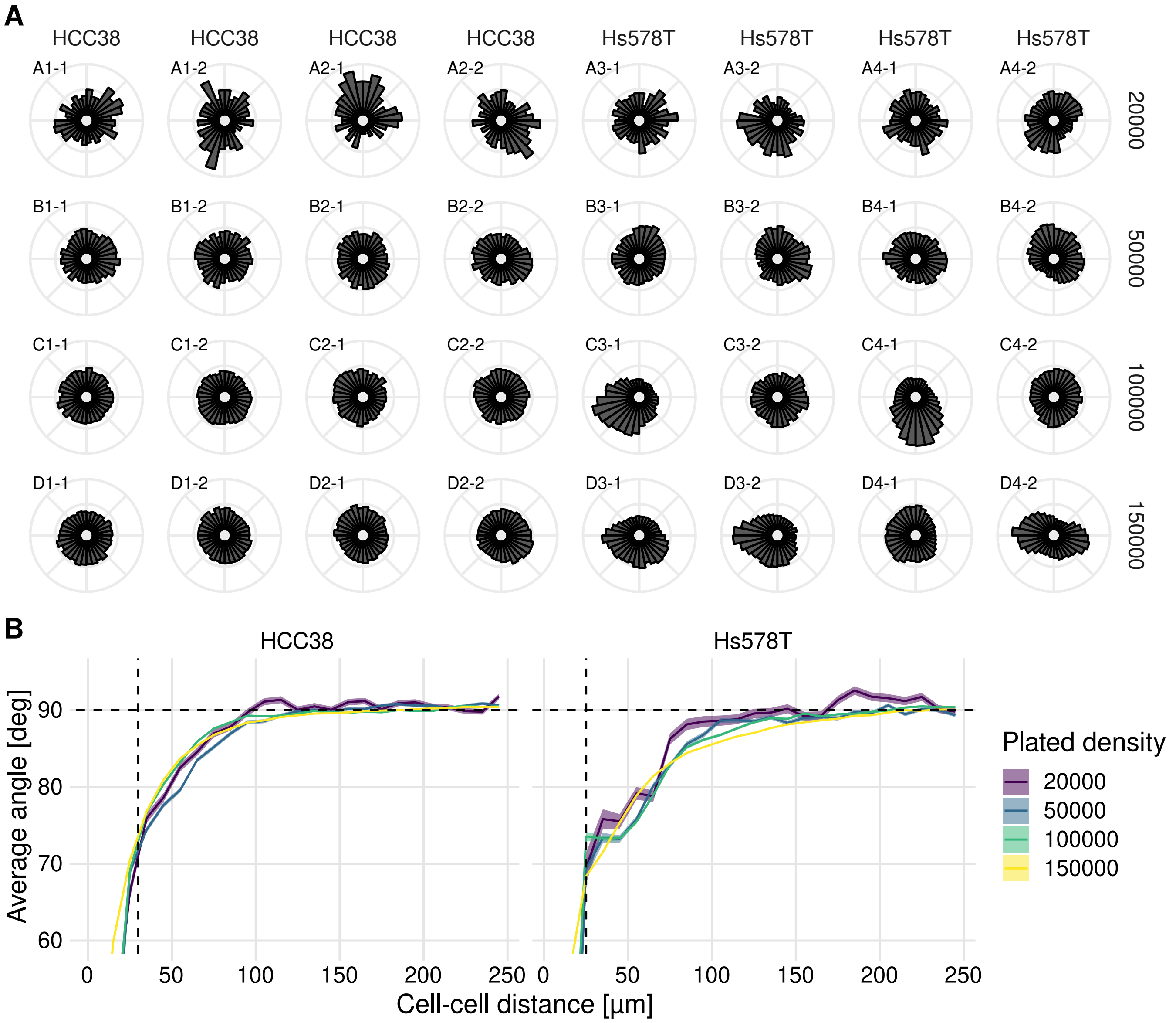

Figure S2. Analysis of large-scale and local streams. (A) Polar histogram of migration directions within a single experiment (24-well plate, see Table 1 for layout). Note that every 2 columns contain technical replicates from the same well, so any potential stage drift should have shown up in at least the technical replicates, but likely also in the whole plate. **(B)** Flow summary with drift correction (subtraction of net overall movement per frame). Horizontal dashed lines denote theoretically expected average angle, vertical dashed lines denote approximate cell diameter.

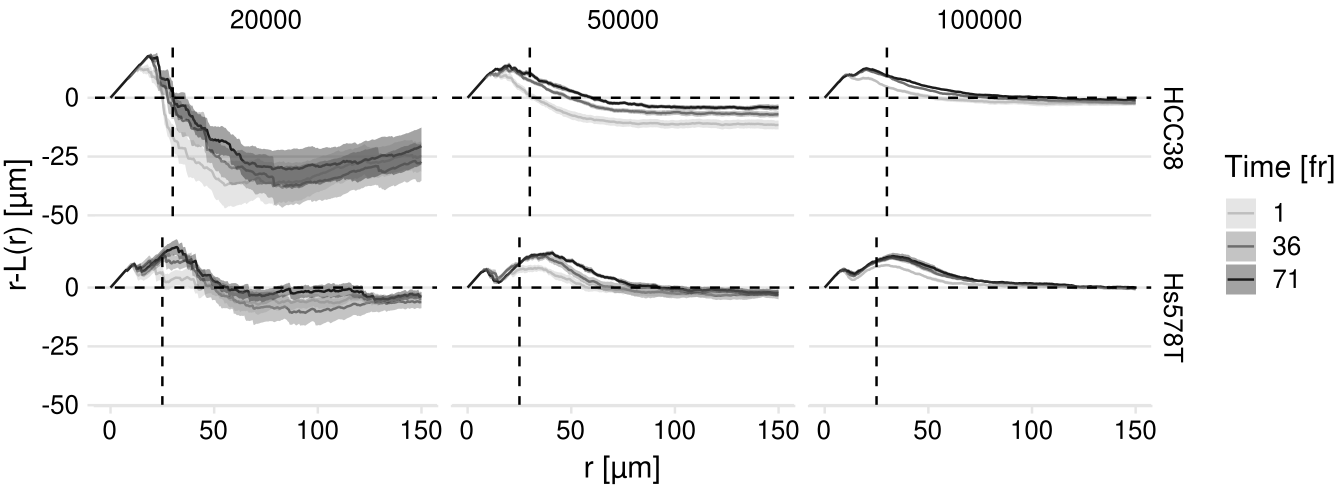

Figure S3. Decrease in clustering over time. The dashed line $r - L(r) = 0$ shows the theoretically expected outcome in case of complete spatial randomness, values above and below this line signify dispersion and clustering. The vertical dashed lines denote approximate cell diameters.

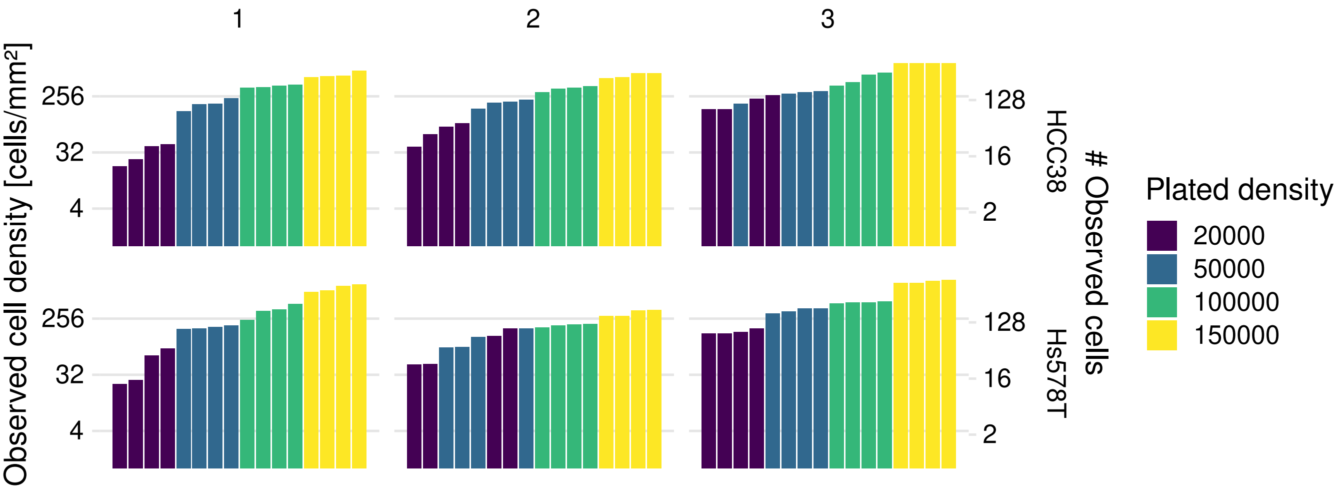

Figure S4. Plated density versus observed cell density in the images for each imaged position. There are 2 wells × 2 positions per condition, and positions are ranked on the number of observed cells. Note that the conditions in Hs578T experiment 2 are not separated well, which is why they were excluded from the Directional Auto Correlation (DAC) analysis summary in which plated density was used as independent variable (Fig. 3B).

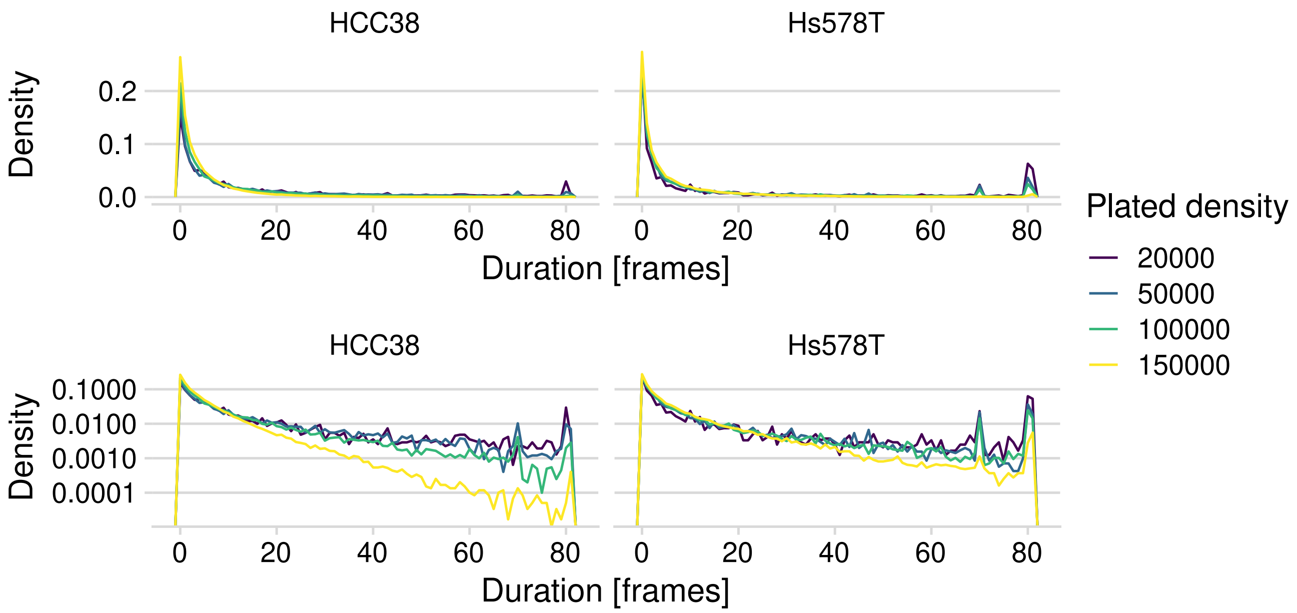

Figure S5. Density plot of track durations. The top panels have a linear vertical axis, and the bottom panels have a logarithmic one. Most tracks are only a few frames long, which is likely due to segmentation and conservative tracking. Especially for HCC38, there are relatively few cells that can be tracked for the complete duration of the experiment. Peaks at 71 and 81 mark the maximum track length for different experiments.

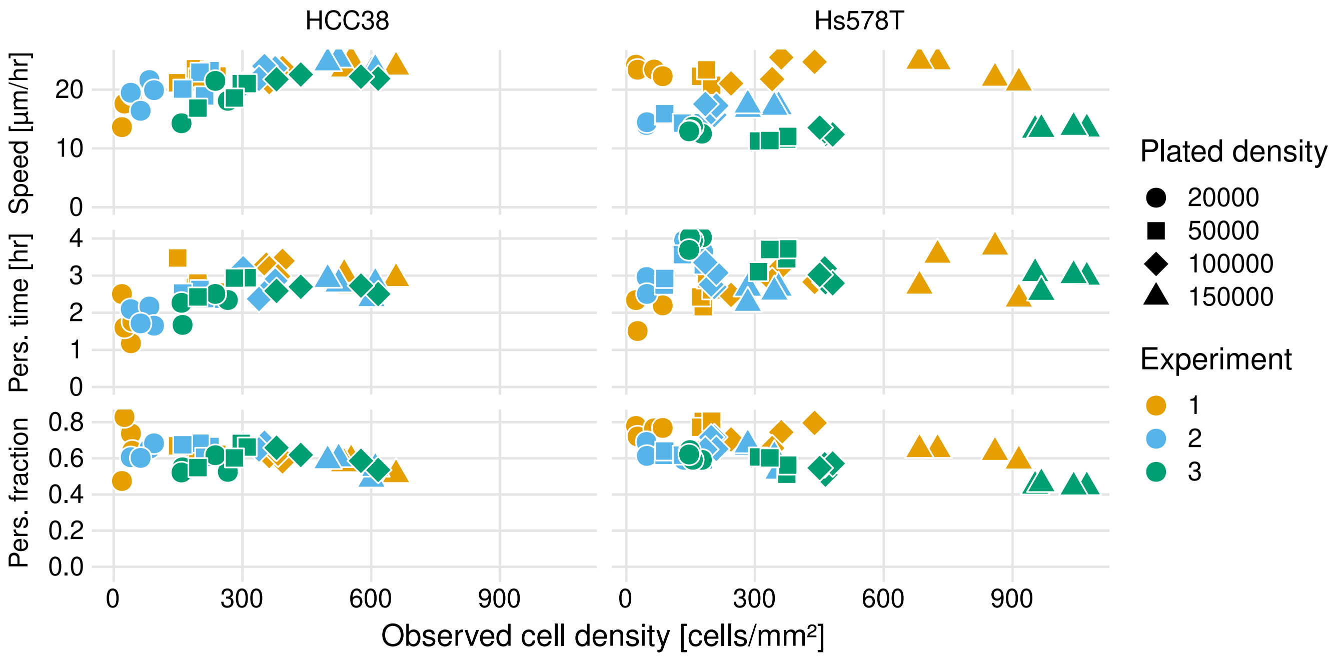

Figure S6. Speed, persistence time, and persistence fraction vs. observed cell density. Estimated parameter values were based on fitting exponential decay of the DAC (see Materials & Methods).

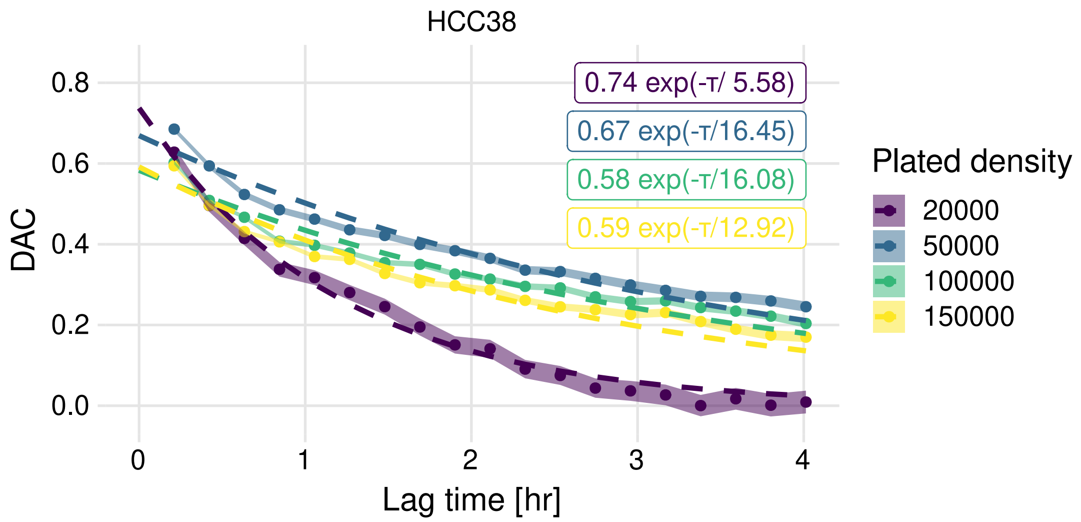

Figure S7. Example DAC fits for all densities of HCC38. The long-run persistence time is fitted well, but the persistent fraction (which is the intersect with the y-axis) is fitted quite poorly.

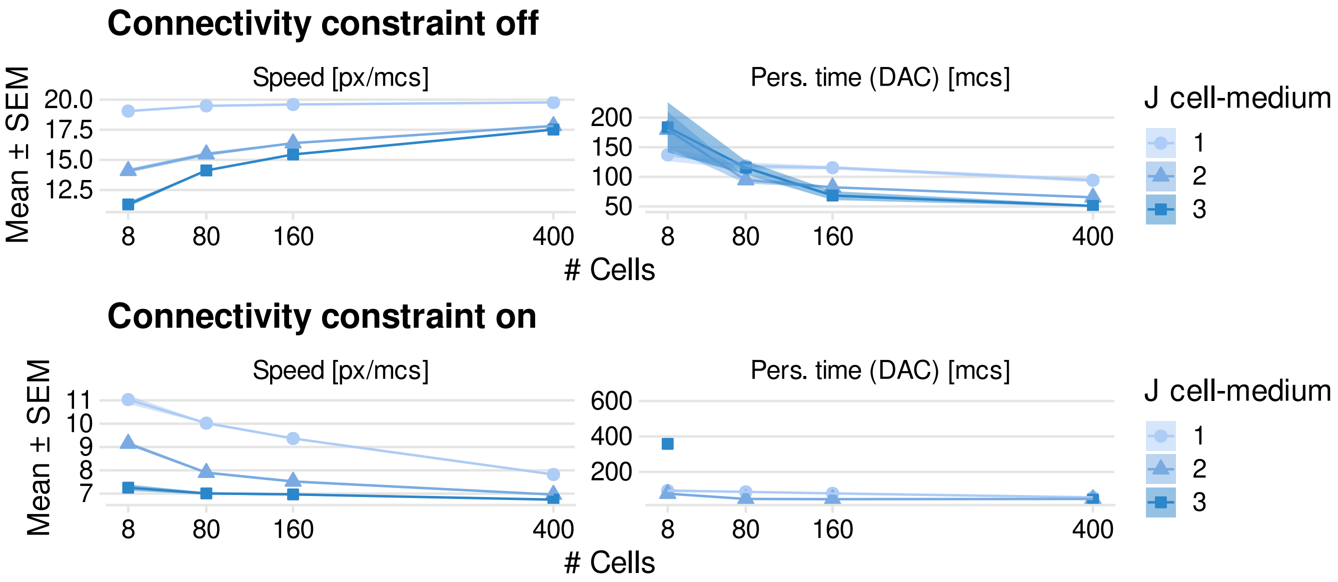

Figure S8. Influence of the connectivity constraint on cellular motility simulated with the basic persistence model using the Morpheus PersistentMotion plugin.

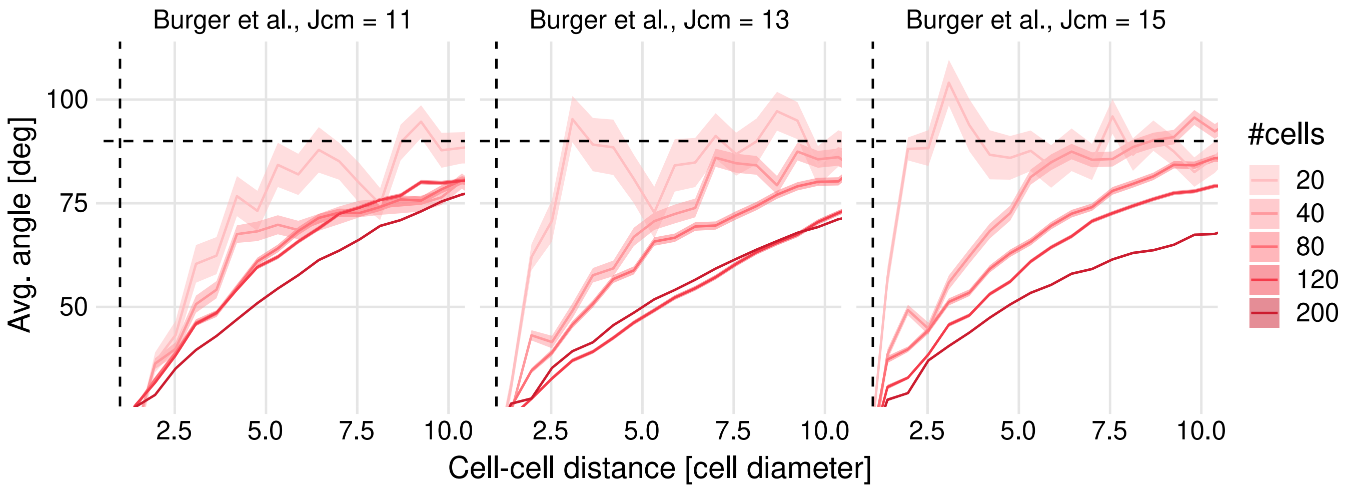

Figure S9. Influence of surface energy $J_{\text{cell,med}}$ and of cell density on stream formation. Results are shown for the proposed persistence model with strong pseudopod coordination.

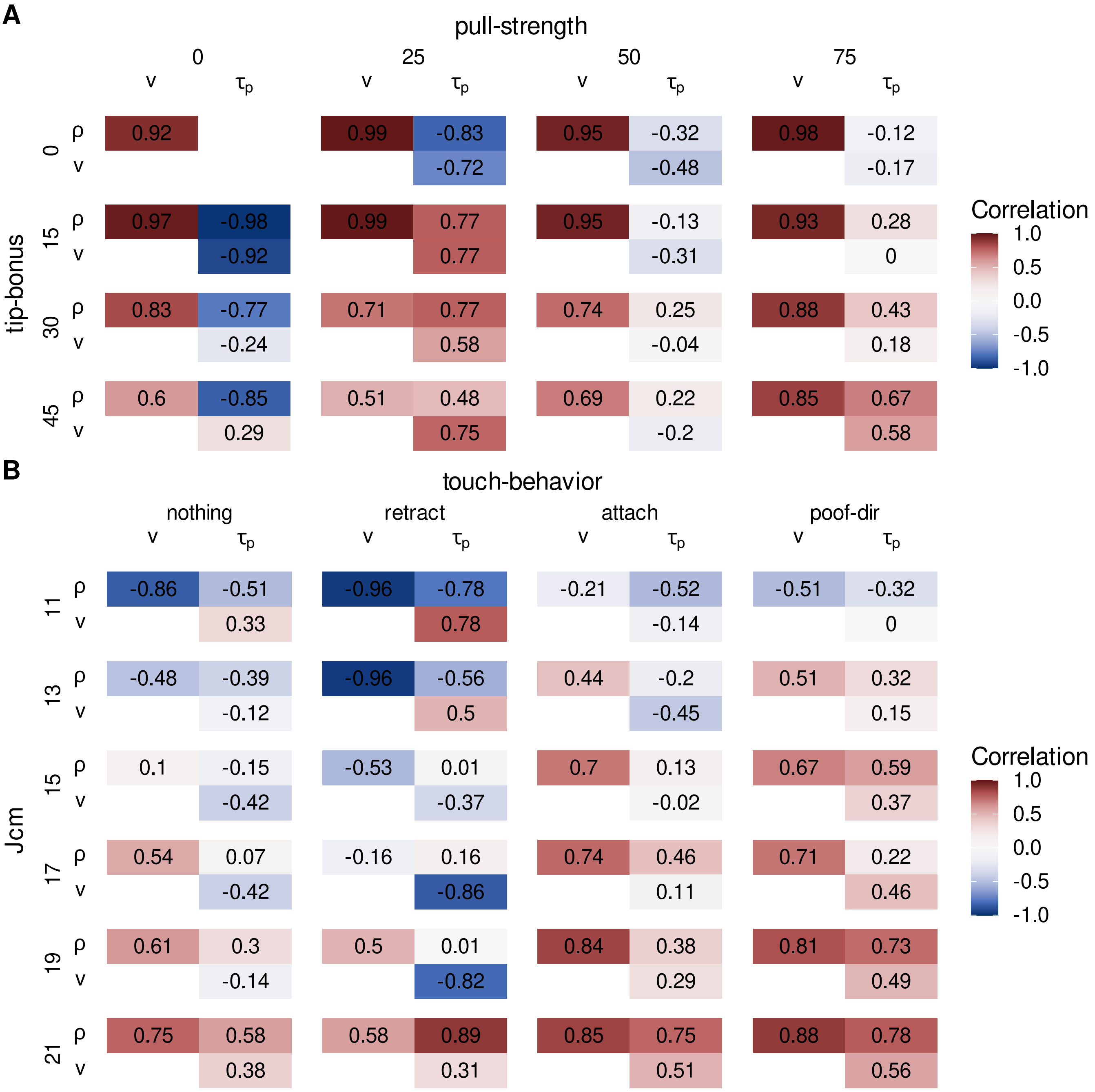

Figure S10. Influence of model additions. **(A-B)** Correlograms for Burger model with **(A)** different values of `tip-bonus` and `pull-strength` at $J_{\text{cell,med}} = 19$ (videos of simulations for `ncells` = 120 can be found in Vid. S6), and **(B)** different `touch-behavior` (`tip-bonus` = 30, `pull-strength` = 50). All correlations are computed with `ncells` = {20, 40, 80, 120, 200} and 3 replicates. Other parameters are the same as in Table S6.

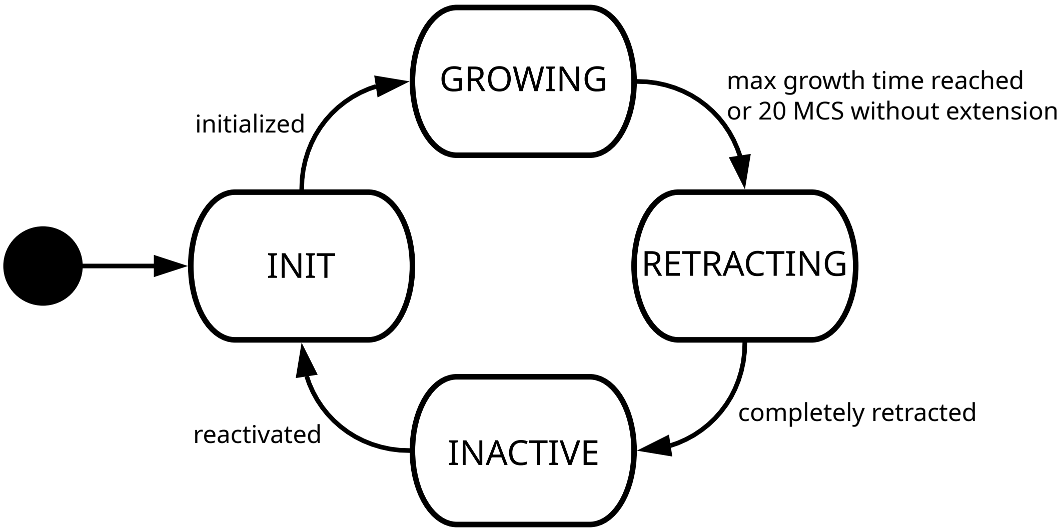

Figure S11. UML state diagram of the pseudopod finite state machine in the Ariotti et al. (2012) model.

1.3 Videos

Video S1. Experimental videos, same wells as in Fig. S1. Also available in higher quality at `https://youtu.be/VFGVDyX_gI4`.

Video S2. Persistence motion with basic persistence implemented in the PersistentMotion plugin in Morpheus. Rows are without (top) and with (bottom) connectivity constraint. $J_{\text{cell,med}} = 3$. Also available in higher quality at `https://youtu.be/lzZJuFTNGC0`.

Video S3. Actin protrusion plugin (Niculescu et al., 2015). $J_{\text{cell,med}} = 4$. Also available in higher quality at `https://youtu.be/TpjjyIsVVgU`.

Video S4. Pseudopod-driven persistence model by Ariotti et al., 2012, implemented in Morpheus. $J_{\text{cell,med}} = 5$. Also available in higher quality at `https://youtu.be/Wn4MP08AJHo`.

Video S5. Proposed persistence model with strong pseudopod coordination. $J_{\text{cell,med}} = 15$. Also available in higher quality at `https://youtu.be/7tpup52ERgo`.

Video S6. Simulation videos for `ncells` = 120 in Fig. S10. Columns show `pull-strength` = {0, 25, 50, 75}, rows show `tip-bonus` = {0, 15, 30, 45}. Also available in higher quality at `https://youtu.be/3CBsb1XGM54`.

REFERENCES

Ariotti, S., Beltman, J. B., Chodaczek, G., Hoekstra, M. E., van Beek, A. E., Gomez-Eerland, R., et al. (2012). Tissue-resident memory CD8+ T cells continuously patrol skin epithelia to quickly recognize local antigen. *Proceedings of the National Academy of Sciences of the United States of America* 109, 19739–19744. doi:10.1073/pnas.1208927109

Niculescu, I., Textor, J., and de Boer, R. J. (2015). Crawling and Gliding: A Computational Model for Shape-Driven Cell Migration. *PLoS computational biology* 11, e1004280. doi:10.1371/journal.pcbi.1004280

Yan, K. and Verbeek, F. J. (2012). Segmentation for High-Throughput Image Analysis: Watershed Masked Clustering. In *Leveraging Applications of Formal Methods, Verification and Validation. Applications and Case Studies*, eds. T. Margaria and B. Steffen (Springer Berlin Heidelberg), Lecture Notes in Computer Science, 25–41. doi:10.1007/978-3-642-34032-1_4
